# Factors influencing health facility delivery among women of reproductive age in Lilongwe District, Malawi: a cross-section study

**DOI:** 10.11604/pamj.2022.43.160.6921

**Published:** 2022-11-29

**Authors:** Samuel Gamah, Henry Oyugi, Richard Muga, Prosper Lutala

**Affiliations:** 1 Clinical Department, Ministry of Health, P. O. Box 30377 Lilongwe 3, Malawi,; 2 Great Lakes University of Kisumu, Kisumu, Kenya,; 3 School of Public Health & Family Medicine, College of Medicine, University of Malawi, Zomba, Malawi

**Keywords:** Factors, facility delivery, reproductive age, Lilongwe, Malawi, maternal health

## Abstract

**Introduction:**

utilization of health facility for delivery could save pregnant women from avoidable maternal deaths. However, use of health facility in deliveries remains inconsistent. The main study objective was to identify factors influencing health facility delivery among women of reproductive age in Lilongwe District.

**Methods:**

a cross-sectional study using structured questionnaires administered to 210 women of reproductive age was used. Chi-square (or Fischer Exact Test), where appropriate was used to analyze data.

**Results:**

the findings of the study revealed that the level of health facility delivery in Lilongwe District is 73.8%. It revealed that 97.1% of the respondents are aware of health facility delivery and most (89.5%) prefer health worker as the best person to assist pregnant women during delivery. There is a significant association between level of knowledge (p = 0.000), the level of education (p=0.000), employment (94.7%) and religious teachings (p=0.000) with delivery in a health facility. The study further shows that more Muslim´s women (91.7%) delivered at health facility compared to their counterparts from others religions and African traditionalists (20%).

**Conclusion:**

in conclusion, the level of knowledge, age, level of education and marital status, Occupation, monthly income, and the amount spent during the past delivery, and the religious teachings influence health facility delivery. We recommend the sharing of the findings with different stakeholders in order to find the solutions and the need for dialogue with traditional chiefs and religious leaders on advocating for health facility delivery.

## Introduction

Maternal health has emerged as global priority because of a great gap in the status of mother´s wellbeing between rich and poor countries. According to the World Health Organization [1], maternal health refers to the health of women during pregnancy, childbirth and the postpartum period. In developed nations where women have access to basic health care, giving birth is a positive and fulfilling experience contrary to poor countries where, giving birth is associated with suffering, ill health and even death.

Internationally, increasing attention given to maternal health has been concentrated in reducing maternal mortality, which is the fifth millennium development goals. Globally, there were an estimated 289 000 maternal deaths in 2013, a decline of 45% from 1990; with the sub-Saharan Africa region alone accounting for 62% (179 000) of global deaths [2]. The global maternal mortality rate (MMR) in 2013 was 210 maternal deaths per 100 000 live births, down from 380 maternal deaths per 100 000 live births in 1990. The MMR in developing regions (230) was 14 times higher than in developed regions [2]. Worse again, alongside countries such as Nigeria, Democratic Republic of Congo, Somalia, Afghanistan, etc. Malawi still has a high MMR which stands at 675/100,000 births [3].

In other words, globally every minute of each year a woman dies from complications of pregnancy, abortion attempts and childbirth [4]. Millions more survive but suffer from illness and disability related to pregnancy and childbirth. It has been estimated that 30 to 50 morbidities occur for each maternal death [5].

Improving maternal health and reducing maternal mortality have been a main concern of several international summits and conferences. It began with the international conference on Safe Motherhood held in 1987 and continued through International Conference on Population and Development (ICPD) 1994 and a review in 1999 (five-year review of the 1994 ICPD) and ended by the Millennium Development Goals [6]. The first conference ended with a declaration calling for a reduction in maternal mortality (at least half) by the year 2000. The ICPD set a goal of reducing maternal mortality to one half of the 1990 levels by 2000 and a further one-half reduction by 2015 [4]. The Millennium Summit in 2000 calls for a 75 percent reduction by 2015 in the maternal mortality ratio from 1990 levels [7]. However, as we beat the deadline, the world was nowhere near achieving this objective, and it is uncertain that the global maternal mortality levels had declined in the past decade to any significant degree [8].

The utilization of health care for delivery is one of the important factors to reduce the incidence of maternal mortality. Data from the developing countries on utilization of maternal services showed that the number of pregnant women who receive at least one antenatal visit is approximately 74 percent in 2005 [7]; 40 percent of deliveries took place in health facilities [4]; and skilled health personnel assisted nearly 61 percent of births in 2006 [7].

World Health Organization [9] has summarized three crucial factors underlying maternal deaths namely lack of access and utilization of essential obstetric services, low socioeconomic status and maternal nutrition. Previous evidences have shown a negative association between maternal mortality rates and health facility delivery. Furthermore, WHO estimates suggest that 88 to 98 percent of all pregnancy-related deaths are avoidable if all women would have to utilise health facility effectively [10]. Access to proper medical attention and hygienic conditions during delivery can reduce the risk of complications and infections that may lead to death or serious illness for the mother and/or baby [11].

In Malawi, utilization of basic maternal health services has remained poor even though there has been increasing public expenditures on the provision of modern health care and training of health professionals. Furthermore, Malawi is experiencing high rates of maternal mortality in spite of the government efforts in improving quality of maternal health services. The maternal mortality rate doubled from 620 per 100,000 live births in 1992 to 1120 per 100,000 live births in 2000; recent estimates show marginal reduction to 984 per 100,000 live birth in 2004 [9] and 807 per 1,000,000 live birth in 2006 [12]. In Malawi, 97 percent of pregnant women attend antenatal (ANC) clinic at least once and only 73% deliver at the health facility by health professionals [13]; figure below the target; which is at 95% of all women attending ANC [14].

Despite the efforts of the government in making delivery health care services free of charge and increasing the human resource capacity; still, pregnant women underutilize health care services during delivery. Hence, this study aimed to investigate factors influencing health facility delivery among women of the reproductive age in Lilongwe. More specifically; this study is looking at several factors that could influence the utilization of maternal health services namely the knowledge level, socio-demographic factors, the level of the facility in the health system delivery, the economic factors, and the cultural factors.

## Methods

**Study design:** the study was a descriptive cross-sectional design.

### Study area

Lilongwe District is located in the Central Region of the Republic of Malawi and bordered by Dedza District in the East, Salima in the North- East and Mchinji District in the western border [15]. Dowa District lies to the north of Lilongwe with Kasungu to its northwestern tip and the Republic of Mozambique to the South West. The total land area is 6159 Square Kilometers representing 6.5 % of Malawi´s total land area. There are 15 traditional authorities and 3 Sub-traditional authorities in Lilongwe District. The Traditional Authority or Sub Traditional Authorities provide the main link between the Local Government and the rural communities. The total population for Lilongwe District was 1,346,360 with females representing 672,506 at the time of data collection. Total number of women in the reproductive ages in Lilongwe was at 301 931 [16]. The district had a total fertility rate of 6.5 above the national´s which stands at 5.7 [17]. Health facility delivery in Lilongwe District and the ANC attendance were respectively at 70.6% and 95% [17]. There were 7 dispensaries, 32 health centers and 5 hospitals in the Lilongwe District. All offered maternity-related services (i.e. normal delivery, assisted-vaginal delivery, caesarean delivery, blood transfusion, etc.) in MOH facilities are free of charge. Chewa was the major tribe in the district. It accounts for 99% of the total population in the rural areas. However, there were three other minor tribes namely the Tumbuka, the Yao and the Ngoni´s. Based on The Poverty Mapping Analysis, 64.3% of Malawi´s population was living in poor households; of these, 63.9% were living in central region and 76.7% of them were for Lilongwe rural [15]. The study was conducted in areas under Sub Traditional Authorities Tsabango and Traditional Authority Chadza in Lilongwe District in Malawi. The 2 areas together have 35,055 women in the reproductive age (15-49). In Sub Traditional Authority Tsabango, there were 7,508 women of reproductive age whilst in Traditional Authority Chadza there were 27,447 women [16]. The reason for selecting these 2 areas was that they present both rural and urban features.

### Population and sampling

The total number of women in the reproductive ages in Lilongwe District was 301 931 (Malawi population and housing Census report, 2008). Inclusion criteria were women of the reproductive age with children below 5 years of age. The study used cluster-sampling technique; 30 by 7 cluster sampling technique. This technique is indicated when it is not possible to obtain a sampling frame [18]. The villages were used as clusters .In Sub Traditional Authority Tsabango there were 29 villages and in Traditional Authority Chadza there were 26 villages making a total of 55. Thirty Villages were selected proportionally, 16 villages from Sub Traditional Authority Tsabango and 14 villages from Traditional Authority Chadza. The following calculations were used to assign villages proportionally:

29 x 30/55 = 16 for Sub Traditional Authority Tsabango and 26 x 30/55 = 14 for Traditional Authority Chadza.

From each village that was selected, 7 women fitting the inclusion criteria were selected through systematic random sampling using a village health registers that record women having children below 5 years from each village. The same village health registers were used to calculate the interval size by dividing the total number of women with children below five years by 7. Using the interval size women were selected to participate in the study.

### Sample size

**Figure 1:**
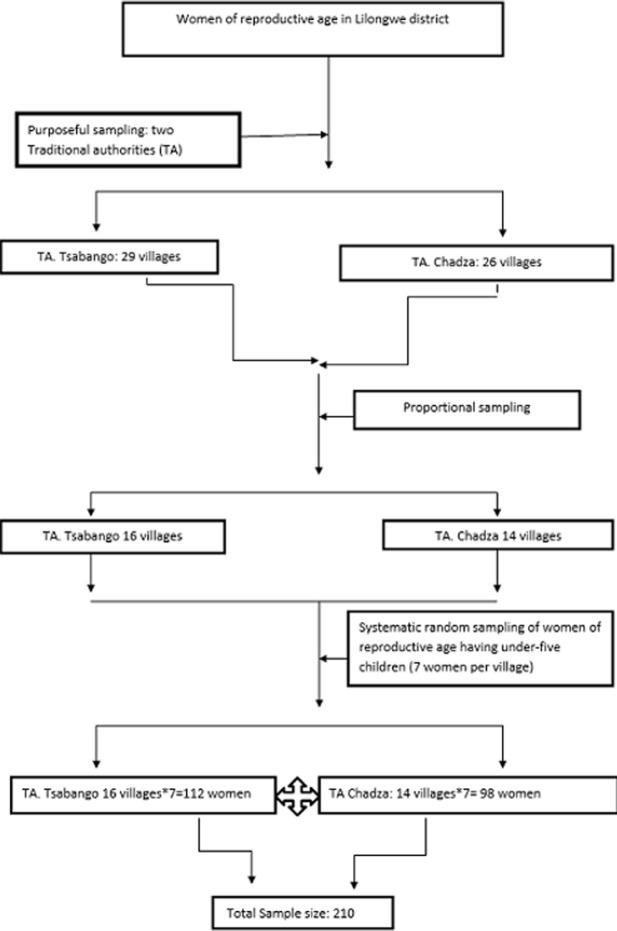
study flow of the sampling

The sample size of 210 respondents was used among women within the reproductive age and having a child below five years of age. The sample was determined by using 30 by 7 cluster sampling and lot quality assessment sampling method [19]. See flow-chart portrayed below in Figure 1.

### Recruitment and selection of supervisors and interviewers

The supervisors with assistance from the researcher selected the interviewers who were fluent in English (passed Malawi School Certificate Exams: secondary certificate) and in the local language (Chichewa) and were employed as Health Surveillance Assistant (community health workers by Malawi government) in the 2 catchment areas. The researcher conducted a one day training for the interviewers on various aspects of the study and a second day was used to pre-test the questionnaire (followed by reformulation of few questions) in under-five clinic at Bwaila Hospital (General Hospital in Lilongwe) on 23 participants.

### Theoretical framework

This study was guided by a ‘Health Services utilization behavioral theory´ developed by Andersen in 1968, then reviewed in 1973 and lastly reviewed in 1997 [20]. The theory states that there are five different approaches that have been used to study the utilization of health care services; the socio-cultural, socio-demographic, social-psychological, organizational and social systems. For the purpose of this study, this theory holds that the level of knowledge, socio-demographic, economic and cultural factors relates to utilization of delivery health care services.

### Data collection

Quantitative data was collected from 210 respondents from 7 February to 21 February 2012 using a structured questionnaire. Structured questionnaire provides data that is objective, scientific and reliable [21]. First step was to identify the household in a cluster (village) where a woman of reproductive age with children below five years stays using the above technique. After ensuring the participants of all ethical considerations, the woman was then requested to choose a place where she will be comfortable to respond to our questionnaire. After the woman gave consent, the field assistant administered the questionnaire and answers were tape-recoded. At the end of each day all the field supervisors reviewed the questionnaire for completeness and accuracy of the data collected before leaving the site.

### Data processing and analysis

Recorded data were sorted, coded, and entered into a computer database. Data were analyzed using the descriptive and inferential statistics. Categorical variables were analyzed using frequencies and percentages. Chi-square test was used to assess the association between independent variables (level of knowledge, socio-demographic, economic, and cultural factors) with the outcome variable. A P-value <0.05 was considered as statistically significant. Data were managed and analyzed using Statistical Package for Social Sciences (SPSS, version 16.).

### Ethical considerations

Permission to carry out the research study was requested and granted by the district health officer, district commissioner, traditional authorities (local administrative and custom officers at village level) and traditional chiefs before conducting the study. Ethical approval was granted by the University of Kisumu Research and Ethical Committee (Ref; GERC/057/1/2012 ) and the National Health Sciences Research Committee in the Ministry of Health in Lilongwe (Malawi) (Approval Number: NHSRC #981).

## Results

### Socio-demographic and economic background

The results (as shown in Table 1) revealed that most of the respondents (75; 35.7%) fall in the age group of 20-24. The highest level of education of most of the respondents was primary education with a representation of 140; 66.1%). Most of the respondents gave birth once (48; 22.9 %), were married (193; 91.9 %), smallholder farmers (116; 55.2%), with a monthly income between 1,500-5,000 Malawian Kwacha (5-16 USD) (41% of participants).

**Table 1:** background characteristics of the respondents

	Frequency	Percent
**Age group(in years)**	**N**	**%**
15-19	15	7.1
20-24	75	35.7
25-29	60	28.6
30-34	49	23.3
=/>35	11	5.2
Marital status		
Single	6	2.9
Married	193	91.9
Widow	2	1
Divorced	9	4.3
Education level		
Primary	140	66.1
Secondary	41	19.8
Tertiary	6	3.3
None	23	10.8
Occupation		
Employed	19	9
Self employed	38	18.1
Farmer	116	55.2
None	37	17.6
Parity of participants		
One	48	22.9
Two	37	22.4
Three	38	18.1
Four	41	19.5
Five and more	36	17.1
Monthly Income ( In MWK)		
None	19	9
1000	29	14
1000-5000	86	41
5000-10000	28	13
10000	12	6
Don’t know	36	17
Total	210	100

N: Frequency; %: percentage; =/>: Equal or superior to; 140 Malawian Kwacha was equivalent to 1 United States Dollar (as of 2012);

### Influence of knowledge of respondents on health facility delivery

The results in Table 2 reveals that most of the participants (97.1%) have ever heard about health facility delivery.

**Table 2:** have you ever heard of health facility delivery

Ever heard of health facility	Frequency	Percent
**Yes**	204	97.1
**No**	6	2.9
**Total**	210	100.0

### Have you ever heard of health facility delivery?

Table 3 shows that 70.4 % of the women who have ever heard of health facility delivery delivered at the health facility and 22.0% delivered at TBA. It further showed that despite information on health facility delivery, a certain percentage continues to delivery at their homes.

**Table 3:** level of knowledge by place of delivery (n=210)

Place of delivery	Ever heard of health facility delivery				Total		p-value†
	Yes		No				
	n	%	n	%	n	%	0.6321
TBA	46	22.0	0	0	46	22.0	
Health facility	148	70.4	7	3.3	155	73.7	
At your home	6	2.9	0	0	6	2.9	
At your parents' home	0	0	0	0	0	0	
Others	3	1.4	0	0	3	1.4	
Total	203	96.7	7	3.3	210	100	

TBAs: Traditional Birth Attendants; **†**Fischer Exact Test

### Level of knowledge

Many participants were knowledgeable of facility delivery concerning its definition, advantages, disadvantages and possible danger sign like vaginal bleeding as a danger sign during deliveries. Table 4 shows that 95.7 have an adequate knowledge (strongly or agreeing with some statements with regard to the above-mentioned points assessing their level of knowledge on health facility deliveries.

**Table 4:** assessment of respondent’s knowledge on health facility delivery

Statement	Strongly agree N (%)	Agree N (%)	Uncertain N (%)	Disagree N (%)	Strongly disagree N (%)
Definition of delivery health care	159 (75.7)	48 (22.9 )	3(1.4 )	0	0
Severe vaginal bleeding is one of the danger signs during delivery	173 (82.4)	36 (17.1)		1 (.5)	0
Advantages of health facility	165 (78.5)	43 (20.5)	1(.5)	1 (.5)	0
Disadvantages of home delivery	159 ( 75.7 )	42 (20.0)		9 (4.3)	0

### Source of information about health facility delivery

Health workers (79%) were major source of health facility delivery than media and other sources altogether.

### Assessment of respondent´s knowledge on health facility delivery

Knowledge was associated with high utilization rate of health facility.

### Factors influencing health facility deliveries

#### Socio-demographic factors

In Table 5 the study revealed that education predicted the likelihood for health facility delivery as it was the case for those who attained secondary (87.2%) and tertiary (83.3%) levels but, taken together the difference was not significant (p= 0.123). The same apply also for pregnant women of low age. In the age ranges of 20-34, health facility-deliveries were higher before dropping at an advanced age. However, this difference remained not statistically different. There was a strong relationship between the Marital status and health facility delivery (p= 0.000). The relationship of respondent´s marital status and place of delivery showed that Divorced women (100%) delivered at health facility more than Married (72.9%), Single (66.7%) and Widow (50%). The difference was statically different (p=0.023). Furthermore, the study showed that parity has a significant association with health facility delivery (p=0.035) with married women delivering more at the health facility.

**Table 5:** influence of socio-demographic factors on place of delivery

Factors	Traditional birth attendant	Health facility	At home and others	Total	P-Values†
**Age ranges**					
15-19	1 (6.7)	14 (93.3)	0	15 (100)	p= 0.272
20-24	16 (21.6)	56 (74.3)	3 (4.1)	75 (100)	
25-29	18 (30.5)	39 (66.1)	2 (3.4)	59 (100)	
30-34	7 (14.3)	38 (77.6)	4 (8.1)	49(100)	
35 and above	4(33.3)	7 (58.3)	1(8.4)	12(100)	
**Education attainment**					
Primary	35 (25.0)	98 (70.0)	7(5.0)	140 (100)	P=0.123
Secondary	5 (12.8)	36 (87.2)	0	41(100)	
Tertiary	0	5 (83.3)	1(16.7)	6(100)	
None	6 (26.1)	15 (65.2)	2(8.7)	23(100)	
**Parity of participants**					
One	9 (18.8)	39 (81.2)	0	48 (100)	P=0.035
Two	7 (14.9)	39 (83.0)	1 (2.1)	47 (100)	
Three	11 (29.0)	26 (68.4)	1(2.6)	38 (100)	
Four	7 (17.0)	31 (75.6)	3 (7.4)	41 (100)	
Five and more	13 (36.1)	19 (52.8)	4 (11.2)	36 (100)	
**Marital status of participants**					
Single	2 (33.3)	4 (66.7)	0	6 (100)	P=0.023
Married	44 (22.9)	140 (72.9)	8 (4.2)	192 (100)	
Divorced	0	10 (100)	0	10 (100)	
Widow	0	1 (50)	1(50)	2 (100)	

† Fischer Exact Test

#### Economic factors influencing facility delivery

There was a strong association between Monthly income of the respondents, occupation, amount spent during the past delivery and health facility delivery and health facility delivery. Respondents with monthly income of more than MWK 10,000 (83.3%) delivered at the health facility more than others with monthly income of less than MWK 1,000 (55.2%) (p=P =0.022). It further showed that women who were employed (94.7%) are more likely to deliver at the health facility than those who were self-employed and farmers (p=0.000). Finally, women who spent more than Malawian Kwacha (MWK) 10,000 (88.9%) during the previous delivery were more likely to deliver at the health facility than those who spent between MWK 1,000 -5,000 (69.4%). However, no any statistically difference was seen among different expenses groups incurred during previous pregnancies (P=0.496) (Table 6).

**Table 6:** economic factors influencing facility delivery

Monthly income					P-values†
Factors	Traditional birth attendant	Health facility	At home and others	Total	P-Values†
None	4 (21.1)	14 (73.7)	1 (5.3)	19 (100)	P =0.022
<1000	7 (24.1)	16 (55.2)	6 (20.6)	29 (100)	
1000-5000	18 (21.0)	66 (76.7)	2 (2.3)	86 (100)	
5000-10000	6 (21.4)	22 (78.6)	0	28 (100)	
>10000	2 (16.7)	10 (83.3)	0	12 (100)	
Don’t know	9 (25.0)	26 (72.2)	1 (2.8)	36 (100)	
**Occupation of the participants**					
Employed	18 (94.7)		1 (5.3)	19 (100)	p =0.000
Self employed	11 (28.9)	27 (71.1)	0	38 (100)	
Farmer	28 (24.2)	80 (69.0)	8 (6.8)	116 (100)	
None	7 (18.9)	29 (78.4)	1 (2.7)	37 (100)	
**Expenses incur during the past delivery**					
Below 500	3 (15)	15 (75.0)	2 (10)	20 (100)	P=0.496
1000-5000	33 (25.1)	91 (69.4)	4 (5.3)	131 (100)	
5000-10000	4 (14.3)	24 (85.7)	0	28 (100)	
>10000	1 (11.1)	8 (88.9)	0	9 (100)	
5 Don't know	5 (22.7)	16 (72.7)	1 (4.5)	22 (100)	

**†**Fischer Exact Test

#### Traditional beliefs about health facility delivery

This study showed that 21(10%) of the respondents have traditional beliefs about health facility delivery against 189 (90%). The participants who have traditional beliefs about facility delivery, further said that the traditional beliefs could influence health delivery by encouraging home delivery by elderly women in the community (47.6%), to allow husband to witness the birth of the child (19%), allowing family to manage themselves the placenta (19%), and expressing the discordance between the government and personal stand regarding the ideal family size (14.4%).

#### Delivery in health facility by religion and ethnicity

Table 7 below shows the Ethnic group and religion of the respondents by place of delivery. It also reveals that respondents from Ngoni tribe (100%) delivered at health facility more than the Yao (92.9). It further shows that 91.7% of respondents who were Muslim delivered at health facility against 75.1% Christians and 20 % African traditionalists. The health facility delivered differs very significantly between religions (p: 0.001); however even if differed between tribes, the difference was not significant (p: 0.070) (Table 7).

**Table 7:** delivery in health facility by religion and ethnicity

Ethnic group of participants					p-values†
**Ethnic group**	TBAs*	**Health facility**	**At Home+ others**	**Total**	
Yao	1 (7.1)	13 (92.9)	0	14 (100)	0.070
Tumbuka	1(9.1)	11 (90.9)	0	12 (100)	
Chewa	43 (26.7)	108 (67.0)	10 (6.3)	161 (100)	
Ngoni	0	14 (100)	0	14 (100)	
Others	1 (11.1)	8 (88.9)	0	9 (100)	
**Religions’ group affiliation of the respondents**					
Christians	39(21.1)	139(75.1)	7(3.8)	185 (100)	0.001
Muslims	1(8.3)	11 (91.7)	0	12 (100)	
African traditions	5(50.0)	2(20.0)	3(30.0)	10 (100)	
Others**	1(33.3)	2(66.7)	0	3 (100)	

**†**Fischer Exact Test

### Level of facility delivery in Lilongwe

According to this study, the use level of health facility in Lilongwe District was at 73.8%.

## Discussion

### Objectives of the study and key findings

This study conducted in Lilongwe, capital city of Malawi aimed to identify factors influencing health facility delivery, to assess the level of knowledge of women on health facility delivery, to identify socio-demographic ( economical, and cultural) factors influencing health facility delivery among women of the reproductive age. Furthermore, the study aimed to determine the level of health facility delivery in Lilongwe. The study revealed a significant association between monthly income, profession, and health facility delivery; age of the respondents, primary or tertiary levels of education, high monthly income, Ngoni tribe, and Muslim religion delivery in a health facility. The level of health facility delivery in Lilongwe stands at 73.8%.

### Strengths and weaknesses of the study

The strength of this study came from the topics itself. Maternal mortality as a proxy of, among others delivery by skilled personnel is attracting attention globally in Malawi and in Africa as a whole. The countdown to the Millennium development goals this year associated with several initiatives of African leaders and development partners to curb the maternal mortality trend can easily explain the importance of results such ours. However, without deleting completely its design, the cross-sectional nature of the study could not allow to get follow-up data. Furthermore, reproductive issues in Africa are very sensitive culturally, and somehow are still discussed in a very respectful and diplomatic way and not openly with a first coming. Quantitative design used here, somehow could not deepen the bottom of some reasons or explain some of the shortfalls of health facility’s users. The contribution on drivers to health facility deliveries of this study is not new in the literature, as it has been recognised by a recent study [22]. It was found that the same drivers identified in small studies were equally the same in the national and even international studies. The value of our study lies in a type of validation of the findings from previous studies in Lilongwe/Malawi context. In addition, the regional estimates are useful as broad indicators of causes of deaths, but national and subnational data are also important to identify differentials due to emerging causes and other local characteristics, such as access to services [23]. Relying on bivariate analysis, our paper is unable to say which variable was strongly associated with facility delivery among outcomes’ variables presenting a statistical significant difference.

### Comparisons to others study

Link between socio-demographic factors and health facility deliveries (Table 5) is not new in the literature. Previous studies [24-30] have demonstrated these associations. Our result about high delivery level in health facility by young women is in line with Kistiana's [28]. This can probably due to high motivation level of young to get healthy babies, which increases their adhesions to medical recommendations. But, one study conducted in Tanzania have shown a high utilization rate of health facilities in women of 25 years above compared to those who were between 16 to 25. This can be explained differently; more likely, that in addition to the age alone, some others confounding could have a strong influence as determinant of health facility use within the age bracket. Local culture, the socially accepted age of marriage, the health seeking behaviour of the general population, and may be the local factors of facility accessibility could play a role in the difference.

The parity shares the same ambivalence with age in relation to health facility delivery. While women with more than 2 deliveries present a low rate of delivering in a facility; a study carried out before; unlike ours, has shown a reverse relation between number of previous pregnancies and the likelihood of facility deliveries. Knowledge in Table 3 is also a factor associated with high utilisation rate of delivery at a health facility. This study agrees with Kistiana [28] findings, which revealed that level of knowledge on health facility delivery increasing the likelihood of delivering in a health facility.

Similarly, this finding agrees with Lubbock *et al*. [29] regarding the source of knowledge. They revealed that their knowledge on pregnancy and delivery practices came from health workers, prior experience, or other more experienced women in the community, especially their mothers and mother´s in-law. This study further revealed; among all the mentioned sources of information that the major remains the health worker (79%). These finding agrees with findings from a study conducted by Kambambai [27], which revealed that health workers advised women who delivered at health unit. The same apply to Malawi, where postnatal care, including routinely education to all postnatal women before they are discharged from maternity to complement the antenatal education given during pregnancy is done systematically in most of the places. The advantages of delivering at a health facility for babies and mothers are one of the pillars of these health talks as well as common postnatal ailments in new-borns and mothers, and alarming signs of complications. Education is tailored to specific needs of each delivered woman is offered individually to address topics such as HIV positive mothers and exposed infants, mothers who went through different procedures (caesarean, episiotomy, etc.), or those who sustain specific others complications, etc.

Contrary to age, the education level seems to correlate with health facility deliveries across different settings and studies [13,25,26]. In Malawi, the Demographic Health Surveillance in 2010 [14] showed that 10% of deliveries from mothers with no education occurred in health facilities compared to 90% of deliveries from mothers with formal education. Furthermore, a study from Ethiopia [26] led to similar results, which showed high utilization rate in secondary schools levels mothers whom the utilization was again higher compared to mothers of primary school level. The demographic Health Surveillance conducted in 2008 in Nigeria [24] confirmed exactly the same results with similar figures to those found in Malawi. The level of understanding, coupled with possible higher socioeconomic level of educated mothers compare to their counterpart could play a role in this use. On the other hand, it has already been shown that occupation increases the likelihood of giving birth in a health facility. Makonnen [26] found the same association regarding the amount spent during the previous deliveries and having an occupation.

Some studies have revealed that cultural factors increases or decreases the likelihood of health facility delivery [13,31]. Tribe of respondents for example has shown a significant difference in facility delivery in Table 7. There is a significant relationship between traditional beliefs and health facility delivery (p = 0.000).Women from the Ngoni tribe (100 %) are more likely to deliver at the health facility more than the Yao (92.9%), Tumbuka (90.9 %) or the Chewa (67.1%). These findings disagree with findings from MDHS, which stated that women from Tumbuka tribe are more likely to deliver at the health facility than other in the country [13]. The proportion of each tribe may be extrapolated to what was found in the MDHS. A study conducted by Seljeskog *et al*. [31] revealed that “in pregnancy, delivery and child care it is the grand-mother , mother and mother in-law who were trusted and perceived knowledgeable and their advices were listened to”. The influence of ethnicity/religion on facility deliveries leads to mixed results. Impact of ethnicity and religion cannot be overemphasized in terms of beliefs, norms and values´ [32]. Staff, decreasing their likelihood to use services, may discriminate against however this relation is not linear and can be influenced by several factors including certain ethnic or religious groups. Studies have shown that Women in some cultures may avoid facility delivery due to cultural requirements such as seclusion in the household during this time of “pollution” [33], specific requirements around delivery position, warmth, or handling of the placenta; or the belief that obstructed labour is due to infidelity [6, 31] ,or of that birth is a test of endurance, and care-seeking a sign of weakness [34-37]. The different patterns of utilization of facility can also explain differences. Moreover, However, ethnicity and religion gave mixed results with regard to health facility delivery; most studies from South America [38-41] showed that indigenous women are less likely to attend skilled delivery. The same applied for ethnic minorities in Asia [42,43] and non-white in South Africa [44]. In Ghana [45] however, no ethnic difference in facility-delivery was detected. Members of the traditional religion and Muslims in Ghana were less likely to use health facility delivery compared to Catholics [32].

According to this study, the level of health facility in Lilongwe District is at 73.8 %. These findings are not far from the findings from Malawi Demographic Health Survey (MDHS 2010) [14] which revealed 70.6% of health facility delivery. With sensitisation of community at the national level and involvement of politicians and traditional leaders on safe motherhood, this figure is expected to rise. For examples in some catchments areas, home delivery is penalising by by-laws and families are expected to pay cash and or some animals. This has played a role in increasing the health facility delivery. On the side of health system also, government with support from development partners has increased the number of nurses and clinicians skilled in reproductive health to counteract with maternal deaths. The regulatory bodies are also awake to watch any breach in the providers-patients relationships to detect any deviation in health professional behaviour, which could erode this effort of all players in the field, even if it is not yet well implanted and monitored.

### Meaning of this study

Facility delivery in pregnant women in Lilongwe doesn´t happen in isolation or by chance, but they are carried or directed by a process associating among others variables such as age of the mother, education, and marital status, etc.

### Unanswered questions and future research

The factors explaining low utilization of health facility despite the fact that more are which traditional beliefs are influencing health facility deliveries. Reason why women still using TBAs for delivery despite their awareness on the advantages importance of delivering in a Health facility, the root reasons beyond low use of facility by women with very low monthly income in a system of free service beyond the quality call for more local exploration. With sensitisation based on the results from this study and others, on factors influencing facility delivery, we will reach a point whereby the services will be overused; which could jeopardise the quality of offered services. Unfortunately, for the moment studies on quality of care and drivers of maternal mortalities as well as neonate´s are still rare but could already fill the gap and prepare the health system to counteract any weakness due to overutilization of reproductive health services. Some factors; such as cultural and religious influence in health facility delivery ´; even if alluded to in this study need further investigations in setting like Lilongwe, Malawi , and Africa as a whole and even Asia; where cultural factors are part of the daily life of individuals. There is also, scarcity of studies in relation to how policies affect facility-delivery. For example, some countries in Africa, including Malawi have instituted by Law forbidding traditional birth attendants to carryout home deliveries or by laws allowing traditional leaders to charge some fees in all home deliveries. Exploring the impact of these laws and the use/non-use and some of their outcomes alongside others related policies would assist in contextualising local decisions supporting or not facility deliveries.

## Conclusion

Cultural, socio-demographic and economic and cultural factors influencing the health facilities deliveries as well as the level of facility delivery in the two sites have been identified. Few implications can be drawn from here: 1) For policy purpose, special guidelines could be developed in these facilities and others locating in similar settings with regards to all factors influencing negatively the facility deliveries such as low income, unemployment, low spending during the previous delivery (ies), belonging to some tribes, to religions others than Christian´s, some age brackets, low or lack of education, etc. these guidelines could directed some interventions such as health talks in reproductive health gatherings, in churches, or in sensitizing different players in matters related to deliveries and safe motherhood. 2) Research has to be conducted to answer to the above unanswered questions which were raised above by this study. 3) In practice, message during health talks and even in churches must emphasise the importance of health facility deliveries and readjust the message to factors, which have been presented in this study. Need for partnership with traditional chiefs and religious leaders on advocating for health facility delivery. To share the findings with different stakeholders in order to find the solutions, improving partnership with Non-governmental organization and relevant institution on how to improve health facility delivery and engaging traditional leaders and religion leaders for a community dialogue to find solutions for the traditional beliefs and religious teachings influencing health facility delivery.

### What is known about this topic

- Facility delivery impacts positively on the mortality of both mother and child;
- Many women in Africa still give birth at home;
- People from resource-limited settings tend to use medical services less than those from higher income countries.

### What this study adds

- Facility delivery is correlated to education, socioeconomic level, and religion;
- The level of facility delivery in Lilongwe was at 73.8%;
- Despite the home delivery, participants still recognize that the health professionals are the most appropriate to go to in case of delivery.
